# Exploration of ANKRD27 as an immune-related prognostic factor in pan-cancer and hepatocellular carcinoma

**DOI:** 10.3389/fonc.2024.1511240

**Published:** 2025-01-06

**Authors:** Ningzhe Shen, Congcong Fan, Haosun Ying, Xinmiao Li, Weizhi Zhang, Jinglu Yu, Jianjian Zheng, Yifei Li

**Affiliations:** Zhejiang Key Laboratory of Intelligent Cancer Biomarker Discovery and Translation, The First Affiliated Hospital of Wenzhou Medical University, Wenzhou, China

**Keywords:** hepatocellular carcinoma, ANKRD27, prognosis, immunity, drug sensitivity

## Abstract

**Introduction:**

Ankyrin repeat domain 27 (ANKRD27) has been found to be associated with certain cancers. However, its clinical potential in pan-cancer remains unclear.

**Methods:**

Public datasets (TCGA and GTEx) were applied to analyze ANKRD27 expression in multiple cancer types and its correlations with immune scores, immune checkpoint genes, and immune modulatory genes. We also examined ANKRD27 expression in hepatocellular carcinoma (HCC) patients using TCGA and GSE14520 datasets. The upregulation of ANKRD27 was verified via qRT-PCR *in vitro*. Based on TCGA-HCC, external, and GSE14520 cohorts, the associations between ANKRD27 expression and survival outcome were explored via the Kaplan-Meier survival curve. The effects of ANKRD27 reduction on HCC cell growth, movement, and invasion were evaluated by CCK-8, Wound healing, and Transwell assays.

**Results:**

ANKRD27 exhibited aberrant expression in multiple cancers and was correlated with immune traits, including immune infiltration, immune checkpoint genes, and immune modulatory genes. Elevated expression of ANKRD27 was found in TCGA-HCC and GSE14520 cohorts and was confirmed in HCC cell lines. HCC patients with high ANKRD27 expression had poorer prognosis. *In vitro*, reducing ANKRD27 decreased the capability of proliferation, migration, and invasion in HCC cells. High ANKRD27 expression was associated with sensitivity to certain drugs.

**Conclusion:**

ANKRD27 displays abnormal levels of expression in different cancer types and is linked to immune status in cancer. Furthermore, ANKRD27 may serve as a prognostic predictor for HCC.

## 1 Introduction

Hepatocellular carcinoma (HCC) is one of the most common malignant tumors globally (1–3). Benefiting from the advancement of therapy methods and drug development, the survival rate of patients diagnosed with HCC has shown a significant improvement (4). Nevertheless, the 5-year recurrence rate among individuals diagnosed with HCC remains alarmingly high at approximately 70%. Additionally, the 5-year survival rate stands at a low rate of less than 10% (5, 6). As such, there is an imminent need to ascertain novel targets or biomarkers for the improvement of diagnosis and prognosis in patients with HCC.

Ankyrin repeat domain 27 (ANKRD27, also known as VARP) has been reported to be implicated in the pathogenesis of esophagitis and respiratory diseases (7–10). Additionally, Mohanad Mohammed et al. demonstrated that ANKRD27 is a risk predictor of colorectal cancer (11). However, a thorough investigation of the value of ANKRD27 in pan-cancer and HCC has not been conducted.

The current research identified abnormal ANKRD27 expression in various cancer types, including HCC. Within TCGA and independent cohorts, there was a compelling correlation between heightened ANKRD27 expression in patients with HCC and an unfavorable prognosis. Additionally, the association between ANKRD27 expression and cancer immunity, immune checkpoints, and anti-tumor medications were explored. Notably, inhibiting ANKRD27 *in vitro* significantly reduces the proliferation, migration, and invasion abilities of HCC cells, highlighting its potential as an oncogenic driver in HCC.

## 2 Materials and methods

### 2.1 Data acquisition

Data of two public pan-cancer cohort (TCGA Pan-Cancer and TCGA TARGET GTEx) were downloaded from UCSC platform (12–14). Specifically, data were selected by removing samples with an expression level of 0 and cancers with less than 3 samples in a single cancer type. A Log_2_ (x+1) transformation was performed on the transcriptome expression values. The GSE14520 dataset was downloaded from GEO. The data of external cohort were collected from a local hospital, which is approved by the First Affiliated Hospital of Wenzhou Medical University (KY2023-198).

### 2.2 Assessing the prognostic value across multiple cancer types

Samples with incomplete survival information and clinical information were excluded. Four types of clinical outcomes (overall survival (OS), disease-specific survival (DSS), disease-free interval (DFI), and progression-free interval (PFI)) were used to assess the prognostic value of ANKRD27 in different cancer types (15). The `coxph` function from the R package ‘survival’ was used in Cox regression analysis (16). The Kaplan-Meier survival curve was utilized to analyze the prognostic differences between HCC patients with high- and low- ANKRD27 expression. The Log-rank test was utilized for statistical assessment to attain significance in prognosis.

### 2.3 Immune traits and RNA modification

Firstly, ANKRD27, a set of 60 genes associated with the immune checkpoint pathway (17), 150 genes related to immune pathways (18), and 44 marker genes for RNA modifications (19) were gathered from a dataset of pan-cancer (20). Then, the stromal scores and the relationship between genes and immune infiltration scores in individual tumors were calculated via “ESTIMATE” and “psych” R packages, respectively (21–23). TIMER 2.0 database provides public immune cell infiltration data for cancers and the infiltration scores of B cell, T cell CD4, T cell CD8, Neutrophil, Macrophage, and DC were measured using the R package”IOBR” (24, 25). The relation between ANKRD27 expression and genes related to immunostimulators, immunoinhibitors, chemokines, chemokine receptors, and MHC was analyzed using the Spearman correlation analysis in R software.

### 2.4 Exploring the relationship between ANKRD27 and clinical features

According to the median value of ANKRD27 expression, the patients with HCC were divided into two groups (high- and low-ANKRD27 expression groups). The prognostic differences between the two groups were analyzed via R package “survival”, and the significance of the prognostic differences between the two groups was assessed using the Log-rank test. The R package ‘pROC’ was used to conduct Receiver Operating Characteristic (ROC) analysis (26).

### 2.5 Functional enrichment

Differentially expressed genes (DEGs) between high- and low-ANKRD27 expression groups were identified by R package “limma” (27) following the criteria: p < 0.05 and |log2-fold change (FC)| > 1.5. Functional enrichment analyses, including Gene Ontology (GO) and Kyoto Encyclopedia of Genes and Genomes (KEGG), were conducted via R package “clusterProfiler” (28). GSEA analysis was performed with GSEA software (29).

### 2.6 Drug sensitivity

Predicted the chemotherapeutic response for each sample based on the largest publicly available pharmacogenomics database (the Genomics of Drug Sensitivity in Cancer, GDSC) (30). The prediction was implemented by R package “oncoPredict” (31).

### 2.7 Cell culture and quantitative real-time PCR

HCC cell lines (Huh-7 and Hep-G2) and normal hepatic cell line (LO2) were cultured with mediums in 5% CO_2_ at 37°C. The cell lines were obtained from the Servicebio company. Total mRNA was extracted with Trizol reagent, followed by reverse transcription into cDNA with Revert Aid First Strand cDNA Synthesis Kit. SYBR Green Master Mix was applied to conduct qRT-PCR, and the relative mRNA levels were commutated via the 2^-ΔΔCT^ method with GAPDH as an internal reference. The primer sequences of ANKRD27 and GAPDH are shown in **Supplementary Table S1**.

### 2.8 Western blotting

The cells were washed in PBS and lysed with RIPA buffer supplemented with a protease inhibitor cocktail. The BCA protein assay kit quantified the protein concentration. Proteins were separated using SDS-PAGE gels and subsequently transferred to PVDF membranes. These membranes were subsequently blocked and subjected to overnight incubation with primary antibodies at 4°C. Following incubation with secondary antibodies, the protein bands were observed and measured using the chemiluminescence detection system and ImageJ software, respectively, with GAPDH serving as the reference standard.

### 2.9 Transfection

ANKRD27-targeted specific small interfering RNA (si-ANKRD27) was synthesized by Genepharma (Suzhou, China). Briefly, cells (6 × 10^4^ cells/well) were added into 6-well plates, incubating at 37°C under 5% CO_2_ conditions. Then, Huh-7 cells were transfected with si-ANKRD27 to knock down ANKRD27 expression following the manufacturer’s protocol. The sequences of si-ANKRD27 are listed in **Supplementary Table S2**.

### 2.10 Cell proliferation, migration, and invasion assays

ANKRD27-knockdown Huh-7 cells were seeded in 96-well culture plates (2 × 10^3^ cells per well). The absorbance at 450 nm was measured daily for three consecutive days using the CCK-8 kit. Transwell chambers were placed into a 24-well plate to detect the invasion ability of the Huh-7 cells with or without si-ANKRD27 transfection. For the wound healing assay, 5 × 10^4^ cells were seeded onto 6-well plates at 48 h after RNA transfection. After reaching 90% - 95% cell fusion, the layer was scratched with a 1000 μl pipette tip. Images were captured using microscopy at 0h and 24h after wounding. All assays were conducted independently in triplicate.

### 2.11 Statistical analysis

The Kruskal-Wallis test was used to assess the gene expression in different tumor tissues. The Wilcoxon rank-sum test was used to compare gene expression between normal and tumor tissues. For comparison analysis, we employed unpaired Student’s t-test and a one-way analysis. Discrepancies in genes mutation frequency across samples was analyzed by the chi-square test. R software or GraphPad Prism was applied in the process of statistical analyses, with significance set at p <0.05.

## 3 Results

### 3.1 Pan-cancer analysis of ANKRD27 gene expression

**Figure 1:**
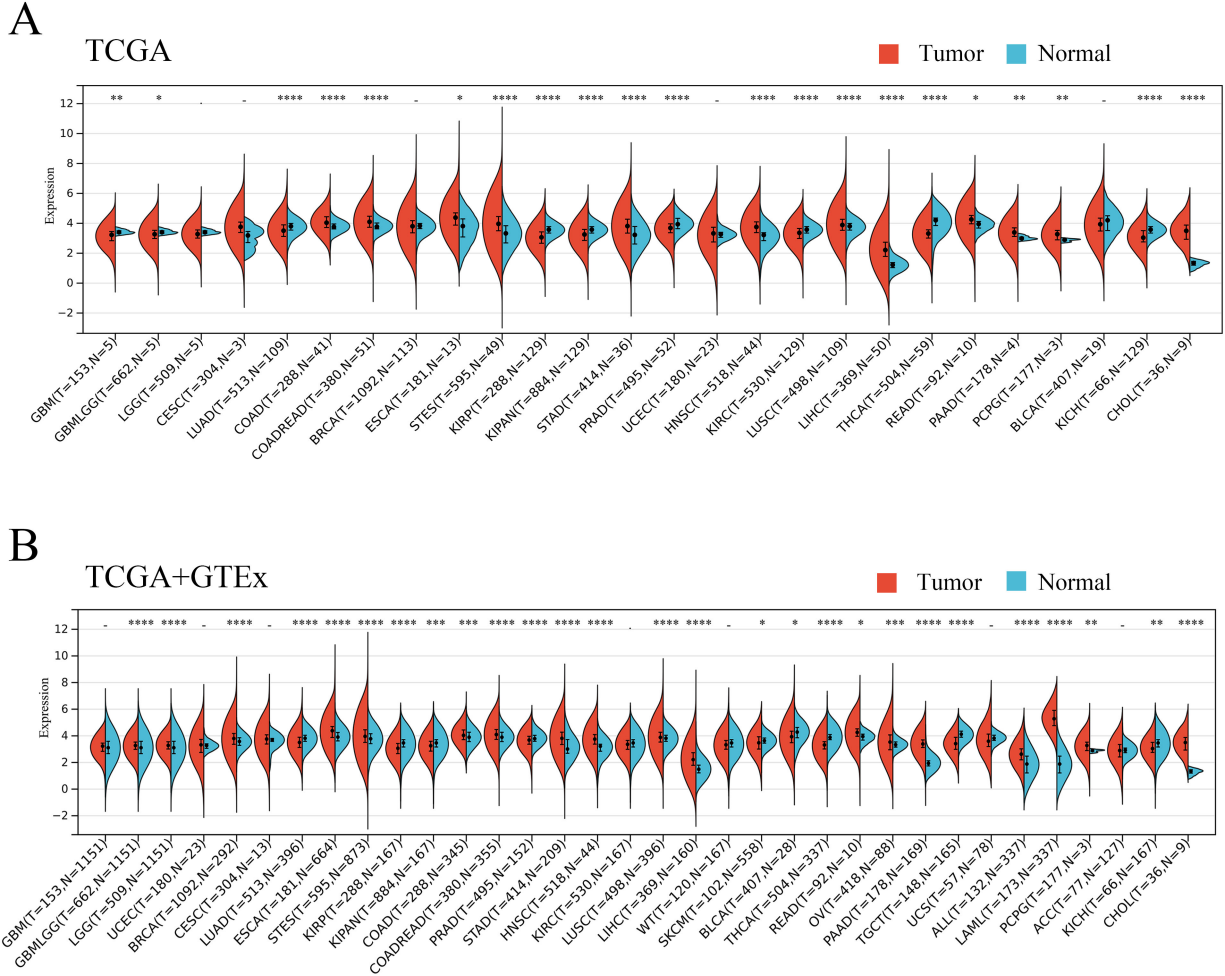
Pan-caner analysis of ANKRD27 expression. **(A)** TCGA. **(B)** TCGA+GTEx. (*p < 0.05, ** p < 0.01, *** p < 0.001, **** p < 0.0001).

To elucidate the expression of ANKRD27 from a pan-cancer perspective, pan-cancer data were collected, encompassing the data derived from the TCGA and GTEx databases. According to the data from TCGA, it was demonstrated that ANKRD27 expression was increased in twelve different cancer types, including LIHC, COAD, ESCA, and others, while significantly lower in nine cancer types, encompassing GBM, LUAD, KIRC, and others (**Figure 1A**). Further, the GTEx database has been introduced to expand the number of normal samples. As shown in **Figure 1B**, ANKRD27 was aberrantly expressed in most cancers (eighteen cancer types), including LIHC, LAML, STAD, and others.

### 3.2 Survival analysis

**Figure 2:**
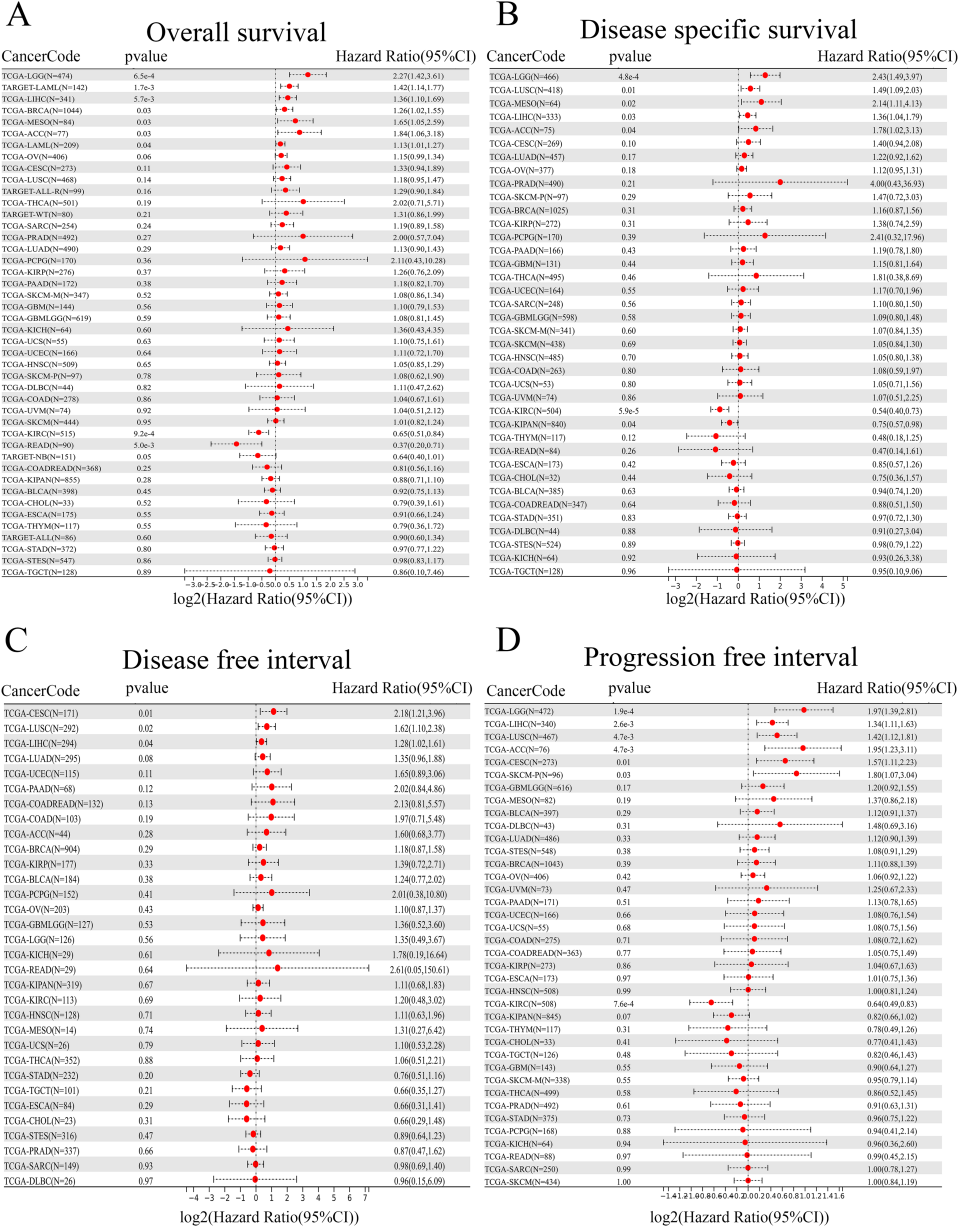
Prognostic value assessment in pan-cancer. **(A)** OS. **(B)** DSS. **(C)** DFI. **(D)** PFI.

To gain insight into the prognostic value of ANKRD27, four types of clinical outcomes (OS, DSS, DFI, and PFI) were analyzed. As shown in **Figure 2A**, heightened ANKRD27 expression emerged as a prognostic risk factor among patients afflicted with LGG, LAML, LIHC, BRCA, MESO, and ACC. Further, a prognostic analysis of DSS substantiated a significant correlation between ANKRD27 expression and five distinct tumor types, namely LGG, LUSC, MESO, LIHC, and ACC (**Figure 2B**). Regarding DFI, it was found that heightened ANKRD27 expression may exert a risk factor among patients diagnosed with CESC, LUSC, and LIHC (**Figure 2C**). Additionally, PFI prognostic analysis indicated that high expression of ANKRD27 was suggestive of a worse outcome for patients afflicted with LGG, LIHC, LUSC, ACC, CESC, and SKCM (**Figure 2D**).

### 3.3 Correlation between ANKRD27 and immune traits

**Figure 3:**
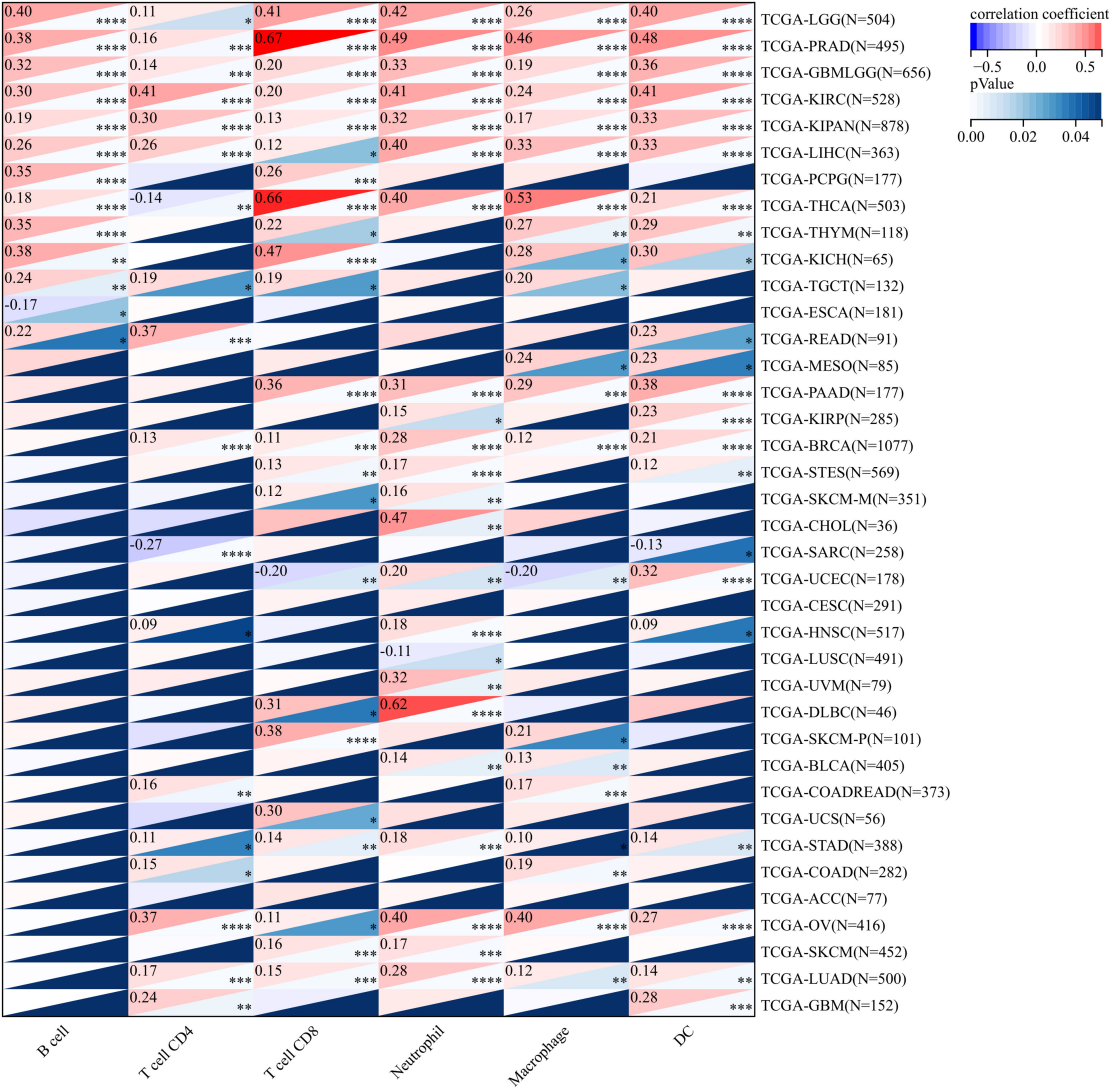
Association between ANKRD27 and immune cell infiltration in pan-caner. (*p < 0.05, ** p < 0.01, *** p < 0.001, **** p < 0.0001).

**Figure 4:**
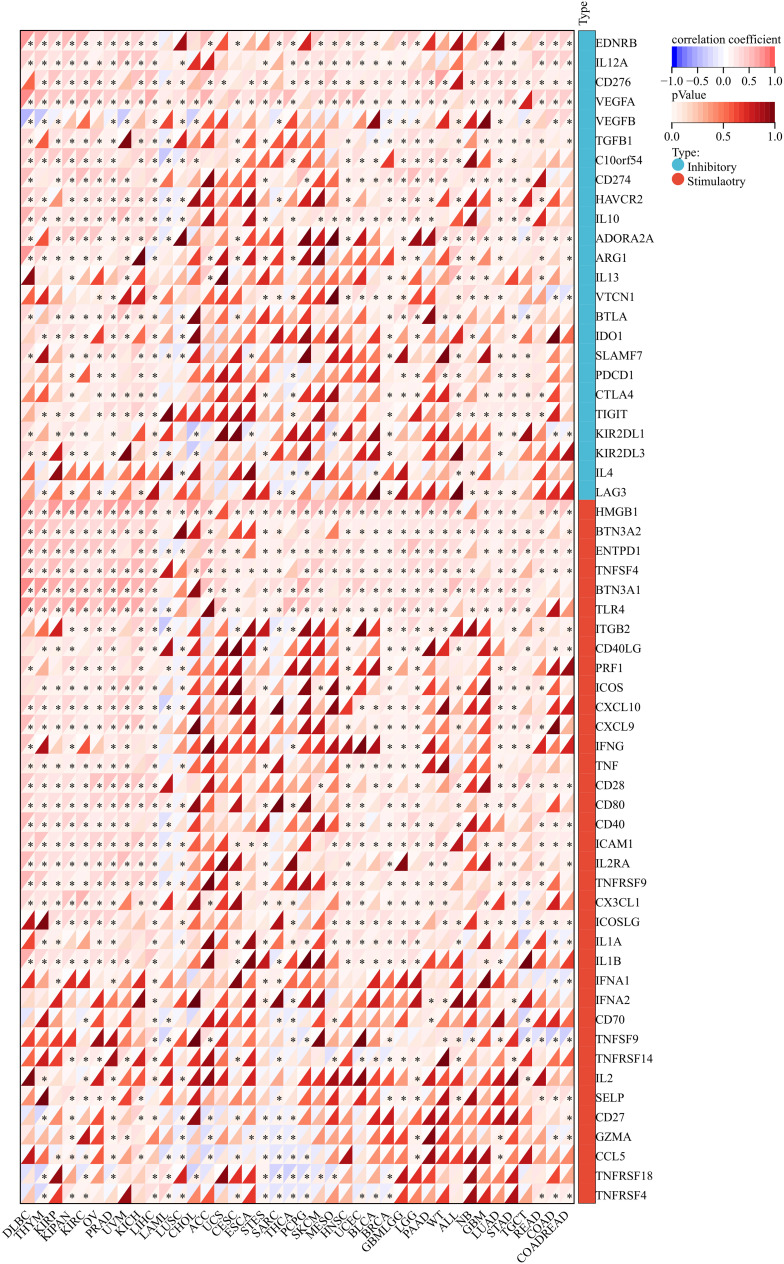
Association between ANKRD27 and immune checkpoints in pan-caner. (*p < 0.05).

In order to gain an in-depth understanding of the impact of ANKRD27 on the tumor microenvironment (TME), we conducted an investigation to examine the correlation between ANKRD27 and the extent of immune traits such as immune cell infiltration, immune checkpoints, immune modulation, and stromal score across diverse cancer types. ANKRD27 exhibited a significant negative correlation with the extent of immune cell infiltration in malignancies such as SARC, LUSC, ESCA, and UCSC, which implied that ANKRD27 might be involved in developing an immunosuppressive microenvironment in cancers (**Figure 3**). Additionally, it was illustrated that ANKRD27 expression showed a positive correlation with the level of inhibitory immune checkpoints, such as CD274, PDCD1, CTLA4, and HAVCR2, across various types of cancer (**Figure 4**). According to the analysis of stromal scores, ANKRD27 expression in GBM, SARC, LUSC, SKCM-P, BRCA, and others was significantly negatively correlated with immune scores (**Supplementary Figure S1**). Additionally, ANKRD27 expression was significantly linked to the levels of various genes related to immunostimulators, immunoinhibitors, chemokines, chemokine receptors, and MHC in a wide range of cancer types (**Supplementary Figure S2**).

### 3.4 RNA modification analysis of ANKRD27 in pan-cancer

Gene expression may be impacted by epigenetics, thus, the correlation between RNA modification and ANKRD27 were explored. Herein, three modification patterns were analyzed, including m1A, m5C, and m6A. **Supplementary Figure S3** showed a strong positive correlation between ANKRD27 and the expressions of different RNA modification-related genes in pan-cancer.

### 3.5 Upregulation of ANKRD27 in HCC

**Figure 5:**
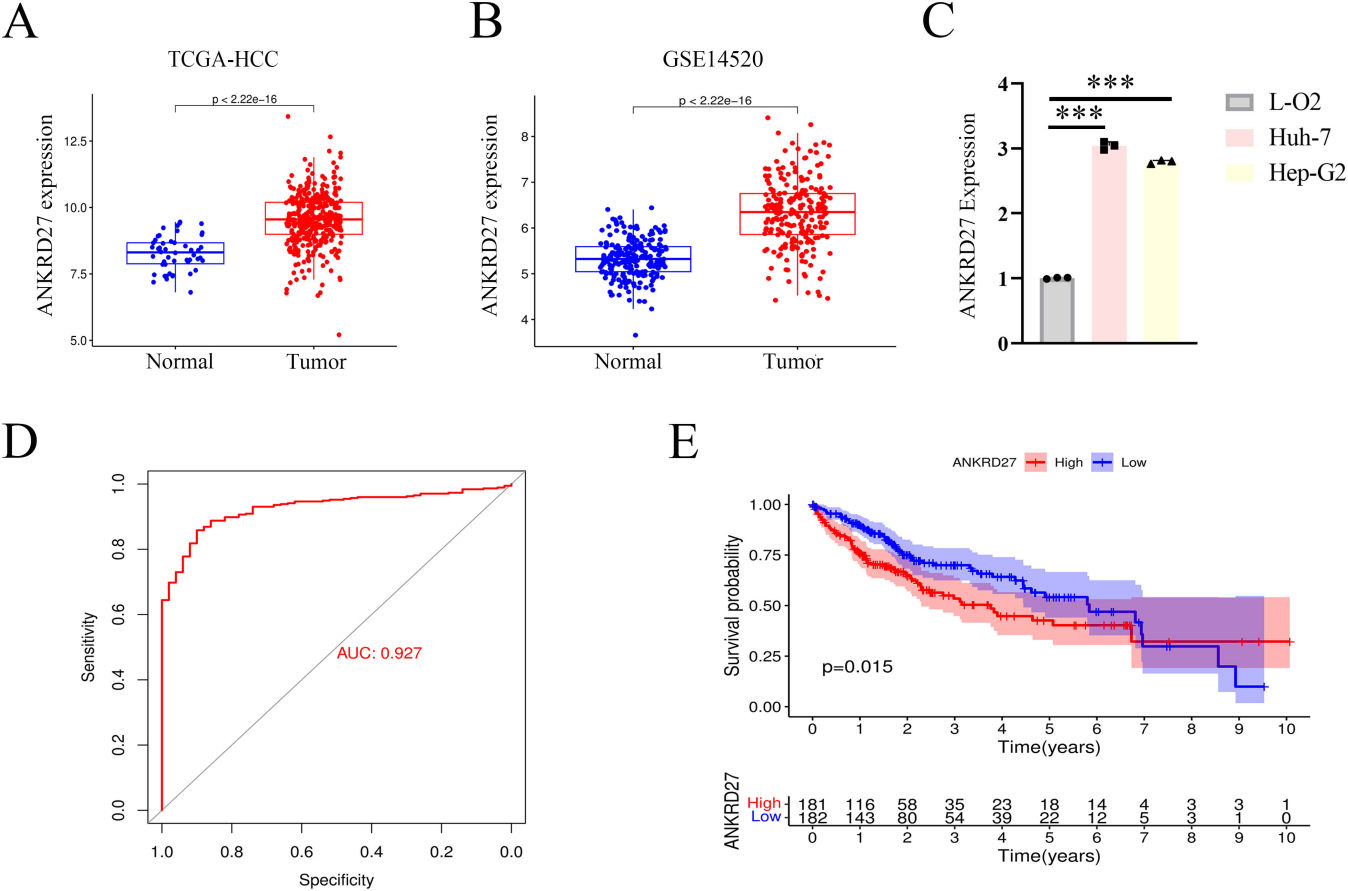
High expression of ANKRD27 and poor prognosis in HCC. **(A)** High expression of ANKRD27 in TCGA-HCC cohort. **(B)** High expression of ANKRD27 in GSE14520 cohort. **(C)** Validation of high ANKRD27 expression in HCC cell lines (Huh-7 and Hep-G2) and normal hepatic cell line (LO2). **(D)** The diagnostic value of ANKRD27 expression in normal individuals and cancer patients. **(E)** The OS survival curve between high- and low- ANKRD27 expression groups. (***p < 0.001).

To assess the role of ANKRD27 in HCC development, we first estimated the expression levels of ANKRD27. In the TCGA and GSE14520 cohorts, there was a high expression of ANKRD27 in TCGA and GSE14520 cohorts (**Figures 5A, B**). Further, the hepatic carcinoma cell lines, Huh-7 and Hep-G2, were utilized to assess the expression levels of ANKRD27, with the LO2 (normal hepatic cell line) serving as a control. The upregulated expression of ANKRD27 was found in Hun-7 and Hep-G2 cell lines compared to LO2 (**Figure 5C**). The results of ROC curve (AUC value = 0.927) suggested the favorable predictive accuracy of ANKRD27 in HCC (**Figure 5D**). Notably, patients exhibiting heightened expression of ANKRD27 demonstrated a significantly diminished OS in comparison to those with lower ANKRD27 expression levels (**Figure 5E**). Additionally, in external cohort and GSE14520 cohort, patients with high ANKRD27 expression showed poorer survival probability as well (**Supplementary Figures S4A, C**). In external cohort, the time-dependent ROC analysis exhibited AUC values of 0.794, 0.614, and 0.636 at 1, 2, and 3 years, respectively (**Supplementary Figure S4B**). In GSE14520 cohort, the AUC values of 1, 2, and 3 years were 0.664, 0.664, and 0.672, respectively (**Supplementary Figure S4D**). The above findings suggested the favorable predictive accuracy of ANKRD27.

### 3.6 Correlation between ANKRD27 and clinicopathological parameters

**Figure 6:**
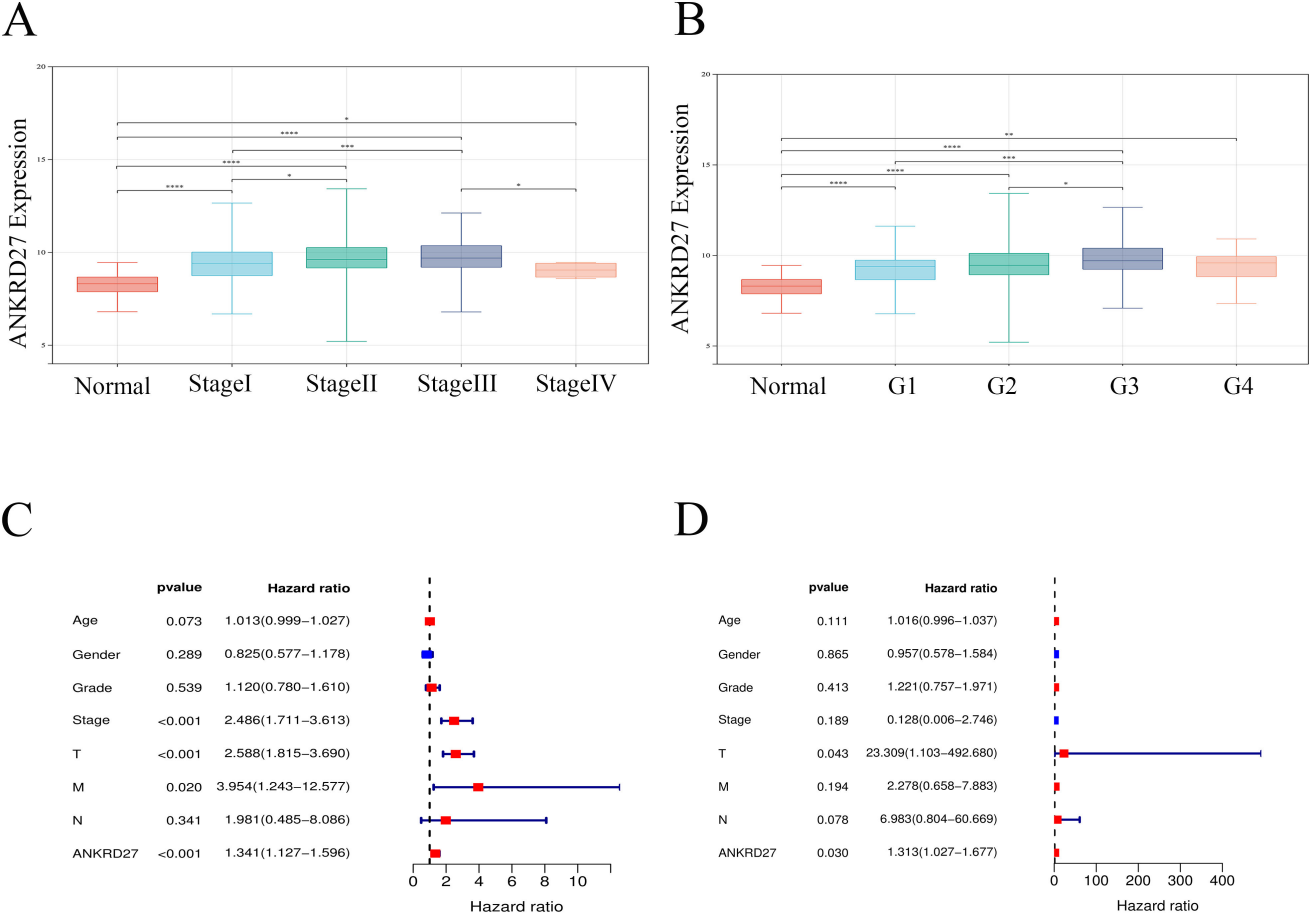
Association between ANKRD27 and clinicopathological characteristics of HCC. **(A, B)** Comparison of ANKRD27 expression in normal liver tissue and cancer tissue with different clinicopathological features, including pathological stage **(A)** and histological grade **(B)**. **(C)** univariate cox regression analysis. **(D)** multivariate cox regression analysis.

Next, to evaluate the potential of ANKRD27 in clinical application, we analyzed the correlation between ANKRD27 and different clinicopathological features (age, sex, TNM stage, histological grade, and pathological stage). The research findings demonstrated that HCC patients with high expression of ANKRD27 exhibited elevated pathological stage, histological grade, and TNM stage compared to the control group (**Figures 6A, B**; **Supplementary Figure S5**). Univariate Cox regression analysis unveiled that ANKRD27 was a prognostic indicator for HCC (**Figure 6C**). Moreover, for patients with HCC, ANKRD27 was identified as an independent risk factor by multivariate Cox regression analysis (**Figure 6D**). These findings underscored the potential of ANKRD27 as a prognostic factor for patients with HCC.

### 3.7 Analysis of functional enrichment and mutation

**Figure 7:**
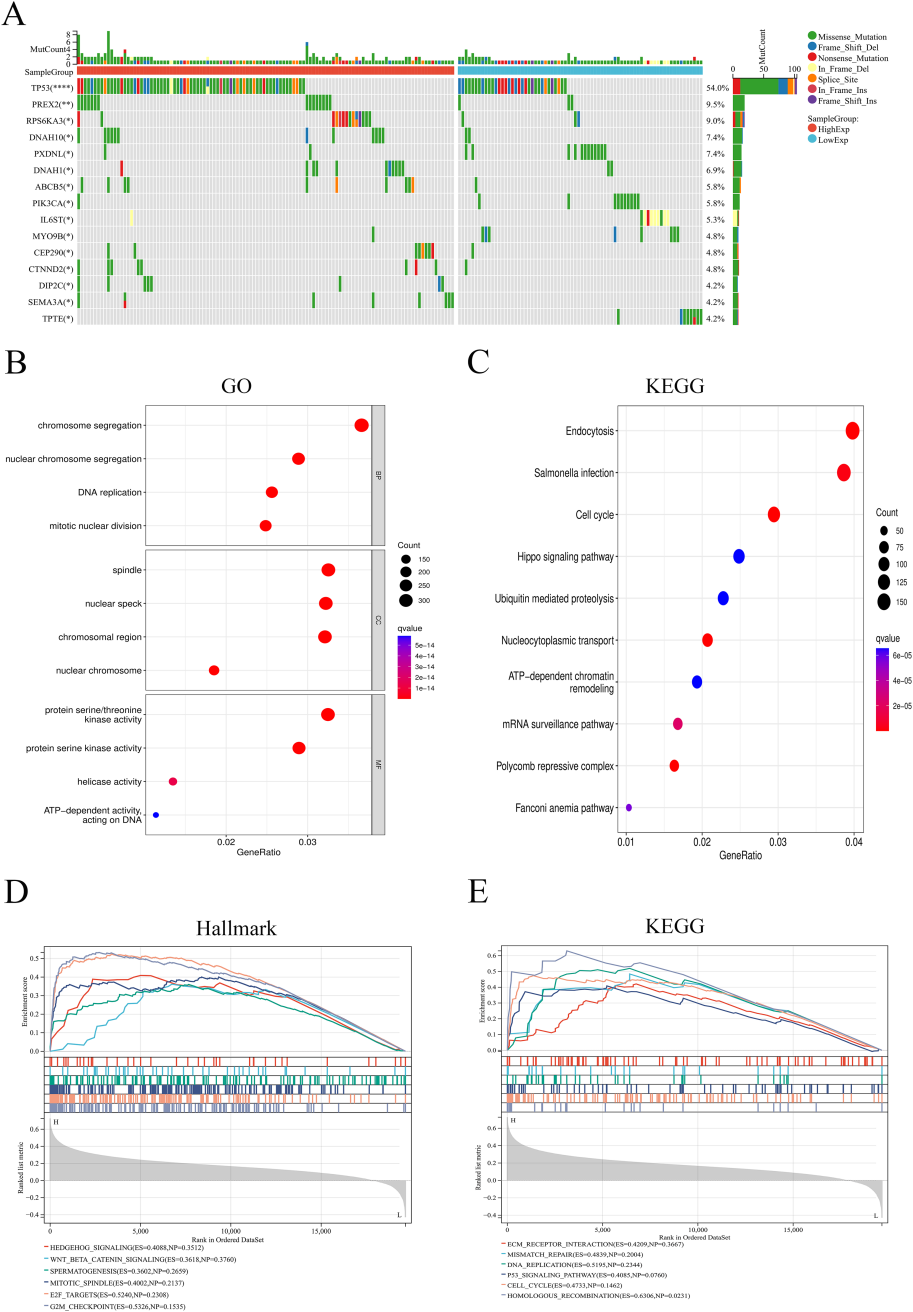
Analysis of somatic mutation and functional enrichment. **(A)** Differences in mutation genes between high- and low- ANKRD27 expression groups. **(B)** Go enrichment. **(C)** KEGG pathway enrichment. **(D)** GSEA analysis in Hallmark gene set. **(E)** GSEA analysis in KEGG gene set. (*p < 0.05, ** p < 0.01, **** p < 0.0001).

Analysis of somatic mutations showed that high expression of ANKRD27 may induce high-frequency mutations in genes such as TP53, PREX2, and RPS6KA3 (**Figure 7A**). The DEGs were used for GO and KEGG functional enrichment analyses. Remarkably, HCC patients with high ANKRD27 expression exhibited a significant enrichment of cancer-related gene sets. GO enrichment analysis revealed that high expression of ANKRD27 enriched in chromosome segregation, spindle, and protein serine/threonine kinase activity (**Figure 7B**). For KEGG analysis, the endocytosis and cell cycle pathways were enriched (**Figure 7C**). The GSEA analysis was further performed. Within the Hallmark gene set, the Wnt/β-Catenin pathway and the G2M_checkpoint pathway was significantly enriched in high-ANKRD27 expression groups (**Figure 7D**). In the KEGG gene set, the homologous recombination and the DNA replication pathway were significantly enriched (**Figure 7E**).

### 3.8 Immune checkpoints and drug sensitivity

**Figure 8:**
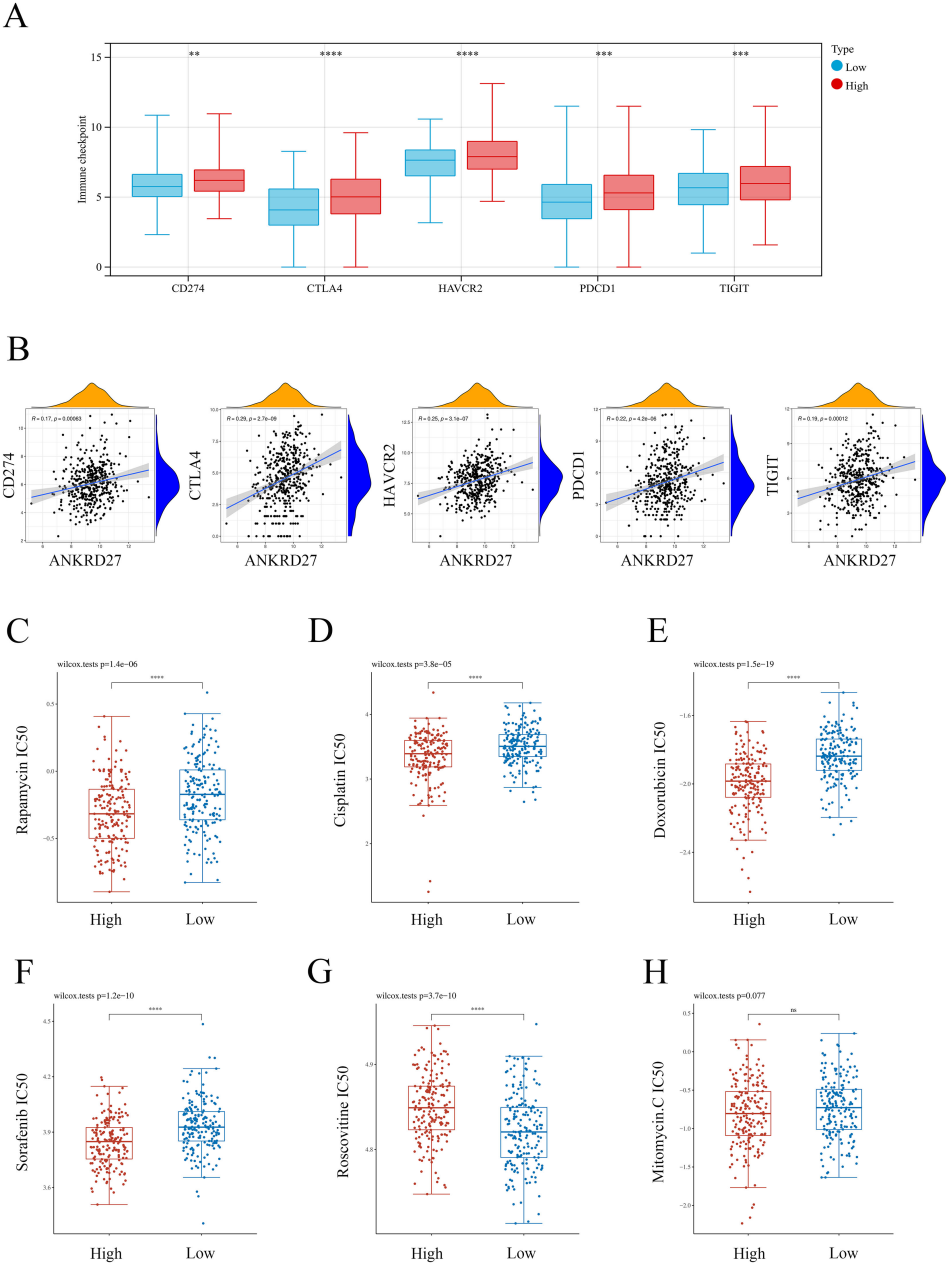
Association between ANKRD27 and immune checkpoints as well as drug sensitivity. **(A)** Comparison of immune checkpoints expression between high- and low- ANKRD27 expression groups. **(B)** Correlation between ANKRD27 and immune checkpoints (CD274, CTLA4, HAVCR2, PDCD1 and TIGIT). **(C)** Rapamycin. **(D)** Cisplatin. **(E)** Doxorubicin. **(F)** Sorafenib. **(G)** Roscovitine. **(H)** Mitomycin. (** p < 0.01, *** p < 0.001, **** p < 0.0001).

Immunotherapy targeting immune checkpoints plays a vital role in the treatment of cancer (32). Thus, we investigated the correlation between ANKRD27 and immune checkpoint molecules. It was found that CD274, CTLA4, HAVCR2, PDCD1 and TIGIT was significantly increased in the high-ANKRD27 expression group (**Figure 8A**). There were positive correlations between ANKRD27 and CD274, CTLA4, HAVCR2, PDCD1 as well as TIGIT in TCGA-HCC cohort and external cohort (**Figure 8B**; **Supplementary Figure S6A**). Furthermore, it was found that the knockdown of ANKRD27 in HCC cells led to a decrease in the expression levels of multiple immune checkpoints (**Supplementary Figure S6B**). These findings suggested that increased ANKRD27 expression may enhance immune escape in HCC.

### 3.9 Drug sensitivity

Rapamycin (33), Cisplatin (34), Doxorubicin (35), Sorafenib (36), Roscovitine (37), and Mitomycin (38) are common chemotherapeutic drugs for patients with HCC. As shown in **Figures 8C-F**, compared to the patients with low ANKRD27 expression, individuals within the high ANKRD27 expression exhibited lower IC50 concentrations of Rapamycin, Cisplatin, Doxorubicin, and Sorafenib. It indicated that patients with elevated ANKRD27 expression may exhibit heightened sensitivity to a range of therapeutic agents, including Rapamycin, Cisplatin, Doxorubicin, and Sorafenib. By contrast, HCC patients with high ANKRD27 expression presented drug resistance on Roscovitine treatment (**Figure 8G**). No notable disparity in drug sensitivity to Mitomycin was observed between the groups characterized by high and low expression of ANKRD27 (**Figure 8H**).

### 3.10 Validation of the role of ANKRD27 in biological behaviours of HCC cells

**Figure 9:**
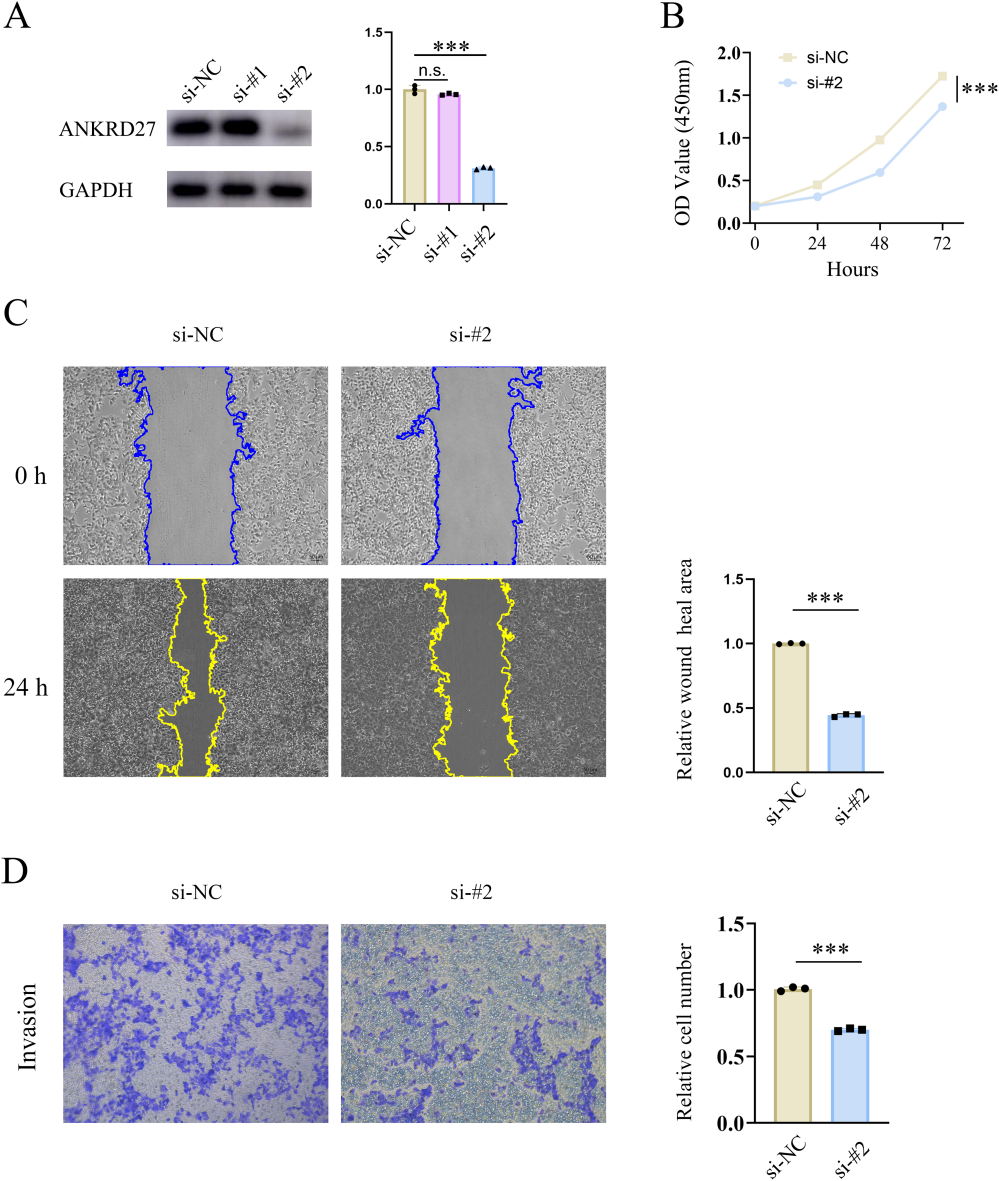
Knockdown of ANKRD27 impeded the cell capability of HCC. **(A)** Knockdown efficiency of si-ANKRD27#1 and si-ANKRD27#2. **(B)** CCK-8 assay displayed the effect of ANKRD27 knockdown on cell proliferation. **(C)** Wound healing assay showed the impairment of cell migration on ANKRD27 knockdown. **(D)** Transwell assay assessed the effect of ANKRD27 on tumor invasion capability. (*** p < 0.001, n.s., no significant).

Furthermore, the capabilities of HCC cells in proliferation, migration, invasion were assessed after ANKRD27 knockdown. Two types of si-RNA targeting ANKRD27 (si-ANKRD27#1 and si-ANKRD27#2) were synthesised. Based on the efficiency of knockdown, si-ANKRD27#2 was selected (**Figure 9A**). ANKRD27 knockdown exerted an inhibitory effect on the proliferative capacity of HCC cells, as evidenced by the CCK-8 assay (**Figure 9B**). Wound healing assay demonstrated that ANKRD27 knockdown attenuated the migratory ability of HCC cells (**Figure 9C**). Additionally, the transwell assay demonstrated that ANKRD27 knockdown significantly reduced the number of cells passing through the wells, suggesting that ANKRD27 knockdown impairs the invasive ability of HCC cells (**Figure 9D**). Thus, the negative effect of ANKRD27 knockdown on HCC cell proliferation, migration, and invasion was confirmed *in vitro*.

## 4 Discussion

Previously, ANKRD27 has been reported to be involved in endosomal transport *in vivo* (39). Moreover, ANKRD27 has been shown to participate in diseases prognosis, including eosinophilic esophagitis, Uveal Melanoma and colorectal cancer (7, 11, 40). In the present study, we conducted assessment of the role of ANKRD27 in pan-cancer. Furthermore, ANKRD27 was demonstrated to serve as a prognostic biomarker and was considered an immune-related factor for HCC.

Expression analysis utilizing data from public datasets (TCGA and GTEx) revealed the dysregulated expression of ANKRD27 in multiple cancer types. Immune responses play a crucial role in the TME (41). We investigated the relationship between ANKRD27 and immune cell infiltration, immune checkpoint expression, stromal scores, and immune modulatory genes at a pan-cancer level. Significant correlations were observed between ANKRD27 and multiple immune features. For instance, high ANKRD27 expression was associated with elevated immune checkpoint expression and poor stromal scores. Recently, the role of RNA modification patterns in tumors, including m1A, m5C, and m6A, has garnered increasing attention (42). m6A, one of the most prevalent mRNA modifications, is involved in a variety of cellular processes, such as RNA transcription, translation, and degradation (43, 44). m5C influences RNA structure and stability, while m1A affects the structure and function of RNA molecules (45). Studies have indicated that these modifications are associated with the prognosis of HCC and play a role in the immune microenvironment (45). In this study, ANKRD27 was found to be associated with RNA modification-related genes suggesting that ANKRD27 may influence gene expression and cellular processes, thereby modulating the progression of tumors.

The specific role of ANKRD27 in HCC were additionally explored. Analysis of two public cohorts (TCGA and GSE14520) revealed aberrant expression of ANKRD27 in HCC. Furthermore, this finding was validated in HCC cell lines (Huh-7 and Hep-G2) *in vitro*. Moreover, upregulation of ANKRD27 was associated with clinical features of HCC, including advanced pathological stage, higher histological grade, and worse TNM stage. Importantly, in both TCGA-HCC cohort, external cohort, and GSE14520 cohort, patients with high ANKRD27 expression exhibited poorer survival probability, suggesting the potential of ANKRD27 as a prognostic biomarker in HCC. Mutation analysis showed a tight association between high ANKRD27 expression and mutations in TP53, RPS6KA3, and PREX2. TP53 is a critical tumor suppressor gene (46), while mutations in RPS6KA3 and PREX2 have been implicated in the development of liver cancer (47, 48). Recent studies have indicated that the expression levels of immune checkpoints may be closely associated with the immunity status of HCC (49, 50). Herein, ANKRD27 expression was found to be positively correlated with the expression of multiple immune checkpoint genes. Moreover, it was found that the knockdown of ANKRD27 led to a decrease in the expression levels of multiple immune checkpoints. These findings suggest that ANKRD27 may participate in the progression of HCC by regulating the expression of immune checkpoints. Drug sensitivity analysis provided insights for therapeutic selection. Notably, patients with high ANKRD27 expression in HCC were found to be more sensitive to treatment with Rapamycin, Cisplatin, Doxorubicin, and Sorafenib. *In vitro* experiments demonstrated that knockdown of ANKRD27 weakened HCC cell proliferation, migration, and invasion, indicating a pro-carcinogenic role of ANKRD27 in HCC. Based on these findings, designing prospective clinical trials to test ANKRD27 as a therapeutic target or biomarker for treatment stratification of HCC patients may be a feasible strategy in future studies.

Indeed, our study has several limitations. Firstly, the majority of the findings are based on publicly available data. Given the limitations of clinical data, which may harbor potential regional or demographic disparities, there is an urgent need for additional prospective data to further validate the prognostic value of ANKRD27 in HCC. Additionally, the specific regulatory mechanisms still require further investigation.

## 5 Conclusion

The comprehensive investigation of ANKRD27 in pan-cancer revealed its prognostic value in cancer. The prognosis, cancer immunity, and drug sensitivity of patients with HCC are linked to abnormal ANKRD27 expression. Furthermore, ANKRD27 may serve as a prognostic predictor for HCC.

## Data Availability

The original contributions presented in the study are included in the article/**Supplementary Material**. Further inquiries can be directed to the corresponding authors.
