# Supplementary material for: Exploration of ANKRD27 as an immune-related prognostic factor in pan-cancer and hepatocellular carcinoma
Source: Front Oncol. 2025 Jan 6;14:1511240. doi: 10.3389/fonc.2024.1511240 (PMC11744007; doi:10.3389/fonc.2024.1511240)
Supplement: Supplementary file 1 [file DataSheet1.docx]

**Supplementary figures**

**
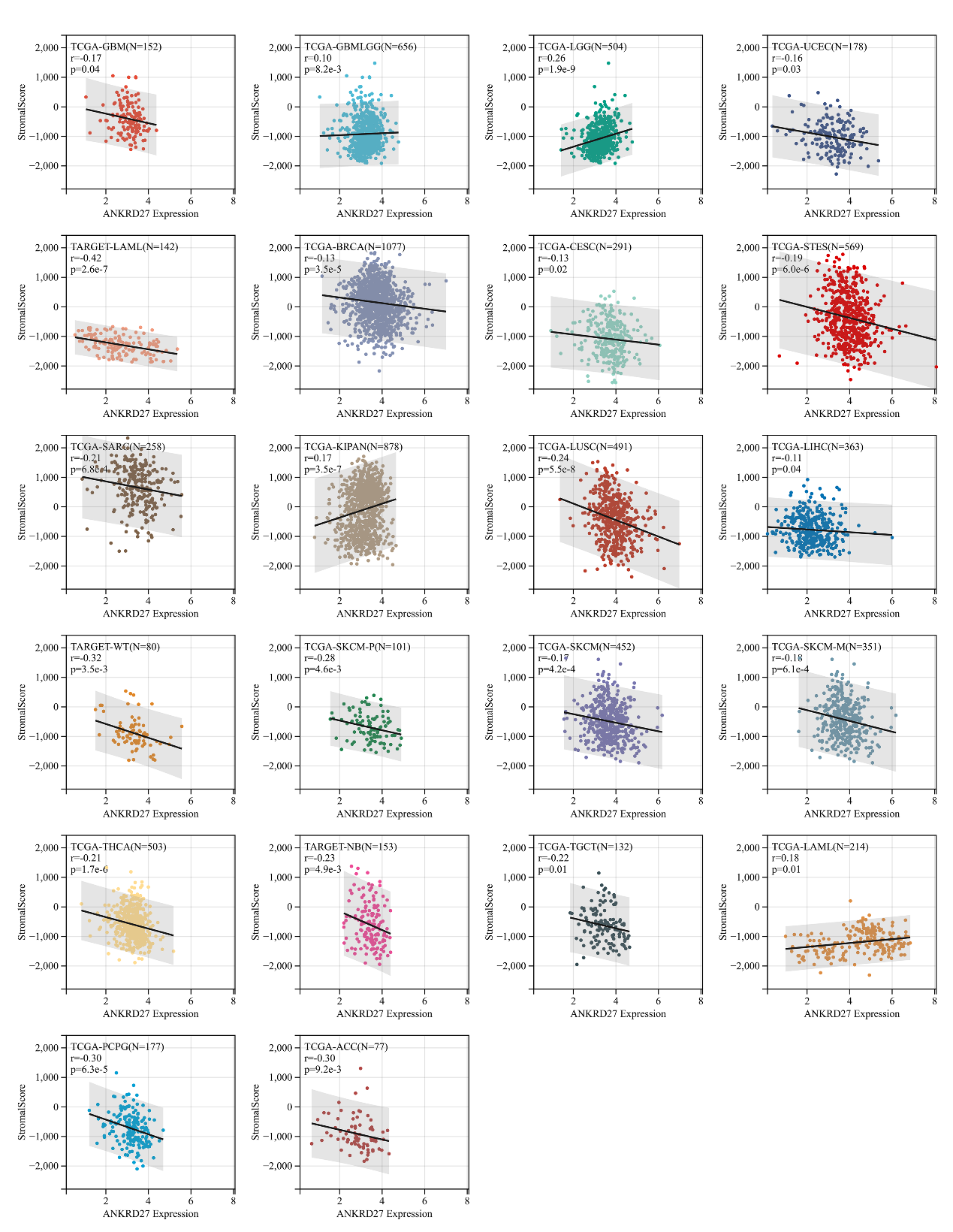
**

**Figure S1. Association between ANKRD27 and** **stromal scores in pan-caner.**

**
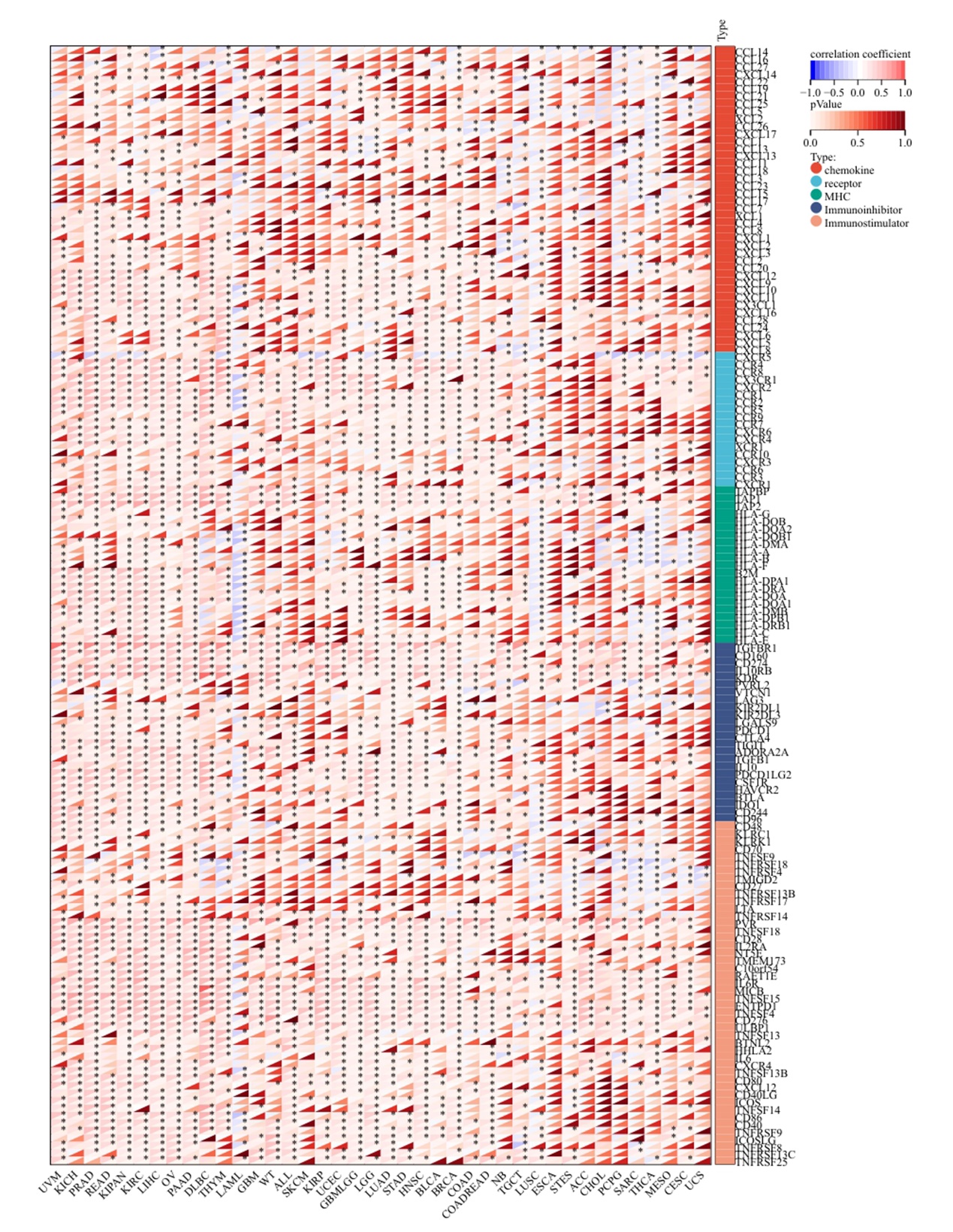
**

**Figure S2. Association between ANKRD27 and** **immune modulatory genes in pan-caner.**


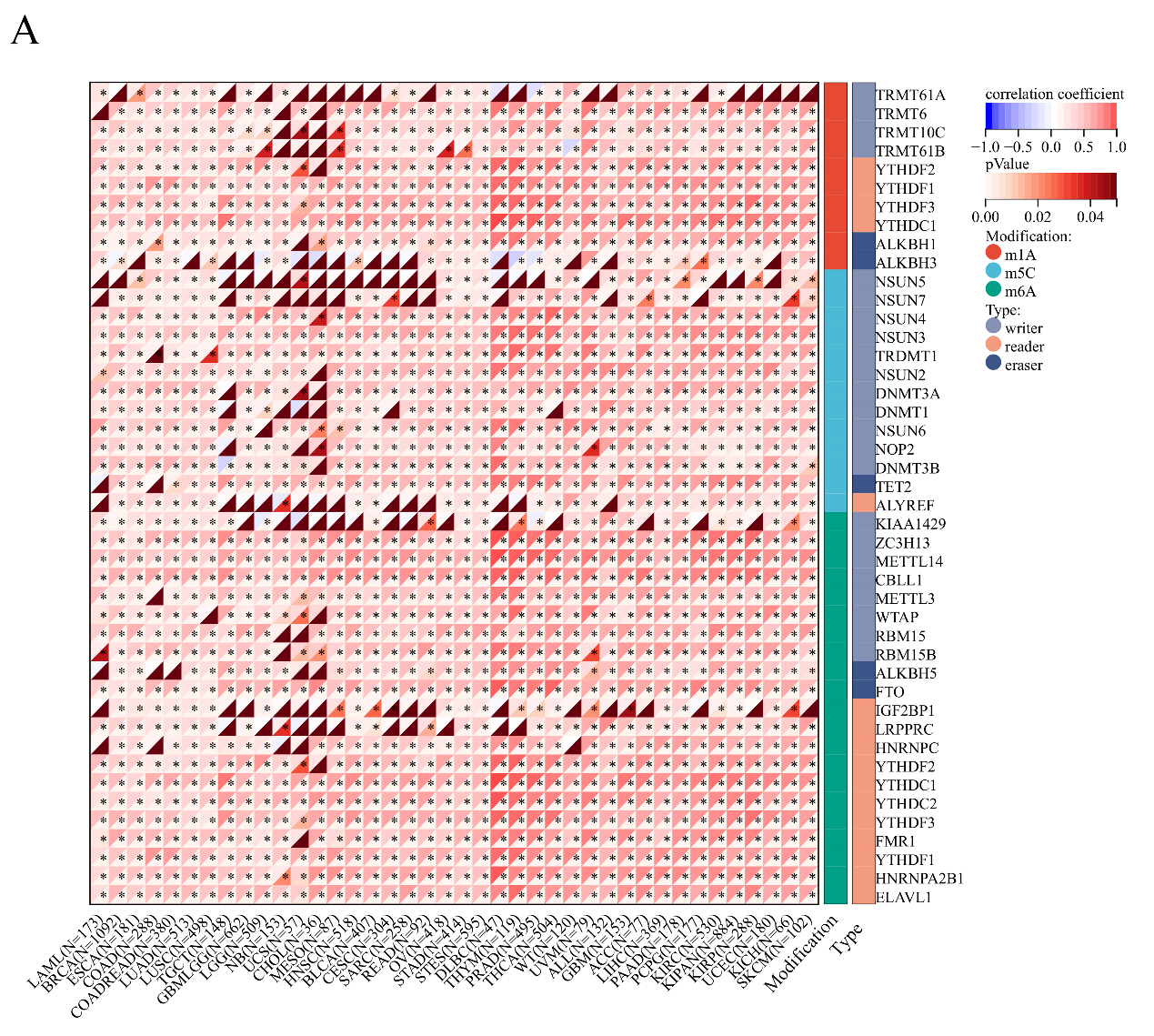


**Figure S3. Association between ANKRD27 and** **RNA modification genes in pan-caner.**


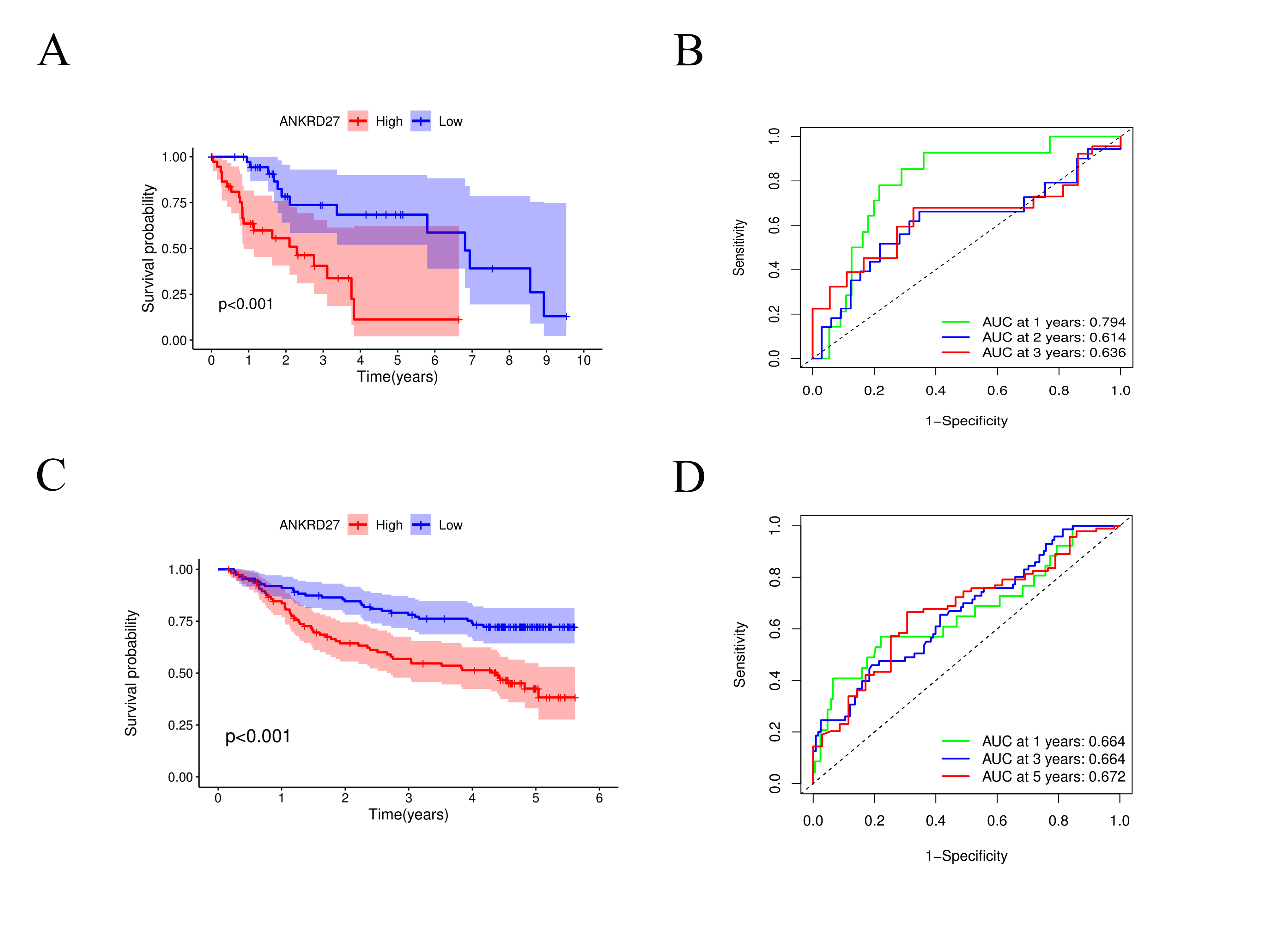


**Figure S4. Prognostic value of ANKRD27 in external cohort and GSE14520 cohort.** (A and C) OS of HCC patients with high- and low-ANKRD27 expression in external cohort (A) and GSE14520 cohort (C). (B and D) Time-dependent ROC curve in external cohort (B) and GSE14520 cohort (D).


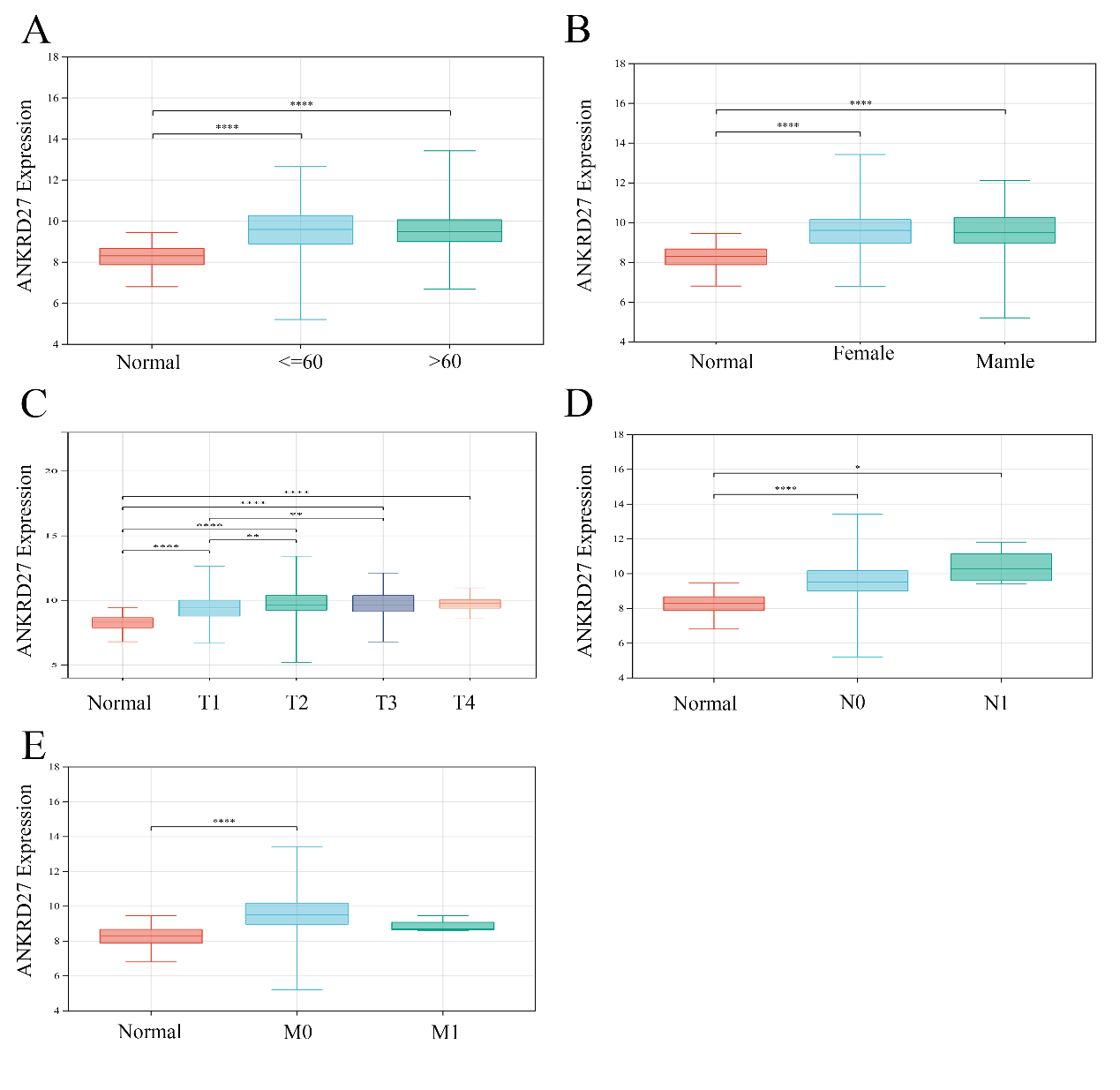


**Figure S5.** **Association between ANKRD27 and clinicopathological characteristics.** (A–B) Comparison of ANKRD27 expression in normal liver tissue and cancer tissue with different clinicopathological features, including age, sex, and TNM stage.


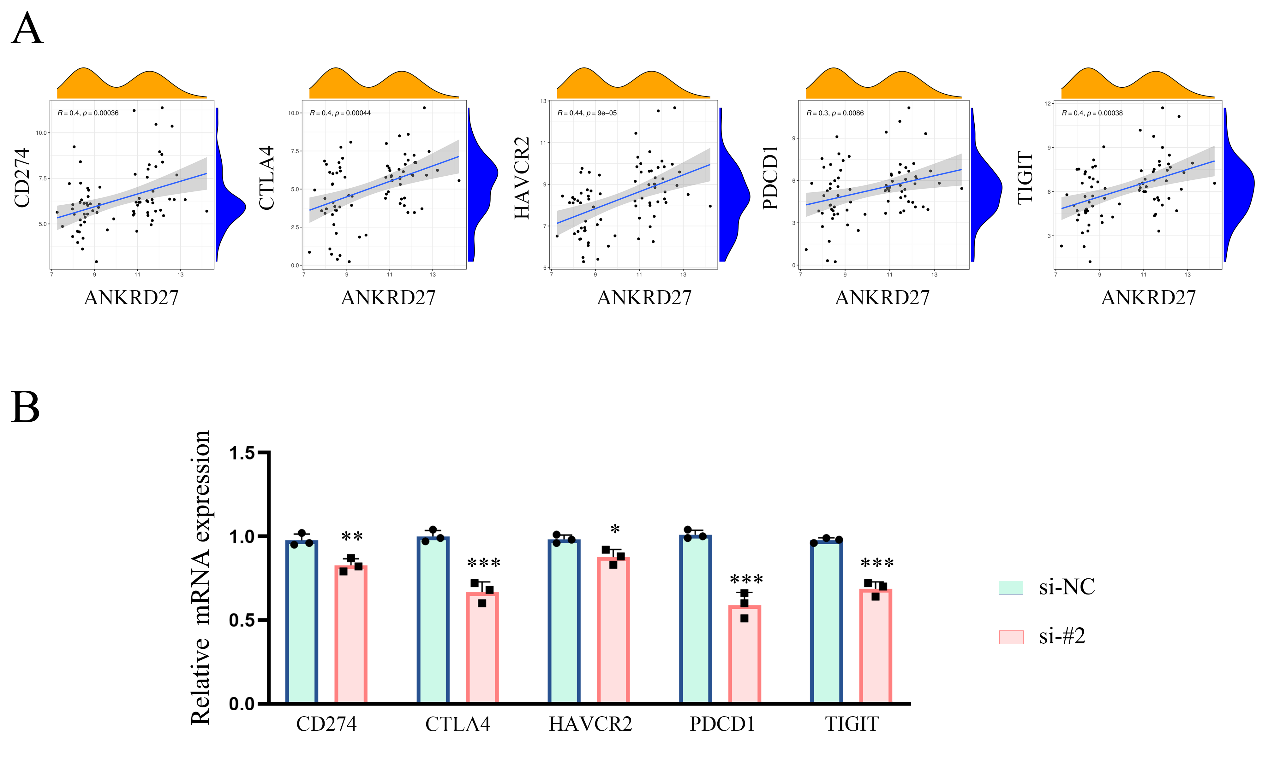


**Figure S6.** **Association between ANKRD27 and immune checkpoints.** (A) Correlation between ANKRD27 and immune checkpoints (CD274, CTLA4, HAVCR2, PDCD1 and TIGIT) in external cohort. (B) The expression of CD274, CTLA4, HAVCR2, PDCD1 and TIGIT after ANKRD27 knockdown.
