# Supplementary material for: Exploration of ANKRD27 as an immune-related prognostic factor in pan-cancer and hepatocellular carcinoma
Source: Front Oncol. 2025 Jan 6;14:1511240. doi: 10.3389/fonc.2024.1511240 (PMC11744007; doi:10.3389/fonc.2024.1511240)
Supplement: Supplementary file 2 [file Table1.docx]

**Table S1. Sequences of primers**

| Gene | Forward sequence | Reverse sequence |
| --- | --- | --- |
| ANKRD27 | 5'-GGACTGTGTGAAGGCTCTGGTTTAC-3' | 5'- CCAGCGGGCAGCAATGTGTAG -3' |
| GAPDH | 5'- AAAGCCTGCCGGTGACTAAC -3' | 5'- GCCCAATACGACCAAATCAGA -3' |

**Table S2 Sequences of si- ANKRD27**

| Gene | Forward sequence | Reverse sequence |
| --- | --- | --- |
| si- ANKRD27 #1 | 5'- CCGGAGUUCAGCUUUAACATT -3' | 5'- UGUUAAAGCUGAACUCCGGTT -3' |
| si- ANKRD27 #2 | 5'- GAGACAGGCUGUUCCUUAATT -3' | 5'- UUAAGGAACAGCCUGUCUCTT -3' |
| si-NC | 5'- UUCUCCGAACGUGUCACGUTT -3 | 5'- ACGUGACACGUUCGGAGAATT -3' |
